# Supplementary material for: Content-rich biological network constructed by mining PubMed abstracts
Source: BMC Bioinformatics. 2004 Oct 8;5:147. doi: 10.1186/1471-2105-5-147 (PMC528731; doi:10.1186/1471-2105-5-147)
Supplement: Additional File 2 — The original results of the above study (non-essential files are deleted to keep the file size under the limit set by BMC bioinformatics). [file 1471-2105-5-147-S2.bz2 › chilibotAdditionalFile2/dip10/393ID8700872E9879/html/FLT4_VEGFC.html]

 


 **FLT4** and **VEGFC** 
  
Found 127 abstracts in PubMed, retrieved 10.  
 

 What does Google say? 
 PDF only 
| .edu only 

---

**Interactive relationship** (e.g. stimulation, inhibition, etc)

**Stimulatory relationship**- VEGF C  [ **VEGFC** ]  promotes intratumoral lymphangiogenesis via VEGFR 3  [ **FLT4** ] , resulting facilitated invasion of cancer cells into the lymphatic vessels.  Ref: 12839674 Zhonghua Bing Li Xue Za Zhi, 2003
- Interestingly, VEGF C  [ **VEGFC** ]  stimulation of LECs induced also the formation of VEGFR 3  [ **FLT4** ]  VEGFR 2 heterodimers, in which VEGFR 3  [ **FLT4** ]  was phosphorylated only at three of the five sites while the two most C terminal tyrosine residues appeared not to be accessible for the VEGFR 2 kinase.  Ref: 12881528 J Biol ChemJ Biol Chem, 2003
**Neutral relationship**- VEGFR 3  [ **FLT4** ]  DC in normal corneas are VEGF C  [ **VEGFC** ]  neuropilin 2, but express VEGF C  [ **VEGFC** ]  in inflammation.  Ref: 12819011 Am J Pathol, 2003
- They belong to the larger family which also includes VEGF, placenta growth factor PlGF and VEGF B, VEGF C  [ **VEGFC** ]  and VEGF D are ligands for the endothelial cell specific tyrosine kinase receptors VEGFR 2 and VEGFR 3  [ **FLT4** ] .  Ref: 12888864 Thromb Haemost, 2003

**Non-interactive relationship** (e.g. studied together, co-existance, homology, etc.)

- Vessels in the parental tumours were found to express VEGFR 3  [ **FLT4** ] , the receptor for VEGF C  [ **VEGFC** ]  and VEGF D.  Ref: 12778077 Br J Cancer, 2003
- OBJECTIVE To investigate the relationship between vascular endothelial growth factor C  [ **VEGFC** ]  VEGF C  [ **VEGFC** ]  expression, VEGFR 3  [ **FLT4** ]  expression, lymphangiogenesis and angiogenesis in human non small cell lung cancer NSCLC.  Ref: 12875690 Chin Med J (Engl), 2003
- We have studied the expression of VEGFR 3  [ **FLT4** ] , its ligand VEGF C  [ **VEGFC** ]  and the co receptor neuropilin 2, in normal and inflamed corneas and characterized the phenotype and distribution of VEGFR 3  [ **FLT4** ]  cells.  Ref: 12819011 Am J Pathol, 2003
- Clinical significance of co expression of VEGF C  [ **VEGFC** ]  and VEGFR 3  [ **FLT4** ]  in non small cell lung cancer.  Ref: 12875690 Chin Med J (Engl), 2003
- OBJECTIVE To study the relationship between angiogenesis and lymphangiogenesis with the expression of vascular endothelial growth factor C  [ **VEGFC** ]  VEGF C  [ **VEGFC** ]  and VEGFR 3  [ **FLT4** ]  in human non small cell lung cancer NSCLC.  Ref: 12839674 Zhonghua Bing Li Xue Za Zhi, 2003
- VEGF C  [ **VEGFC** ]  expression and VEGFR 3  [ **FLT4** ]  expression were positively associated with lymph node metastasis and lymphatic invasion P <.  Ref: 12875690 Chin Med J (Engl), 2003
- Expression of VEGF C  [ **VEGFC** ]  and VEGFR 3  [ **FLT4** ]  was significantly associated with lymph node metastasis P = 0.008 and P = 0.013 respectively and lymphatic invasion P = 0.027 and P = 0.020 respectively.  Ref: 12839674 Zhonghua Bing Li Xue Za Zhi, 2003
- A significant positive correlation was found between VEGF C  [ **VEGFC** ]  in cancer cells and VEGFR 3  [ **FLT4** ]  in lymphatic endothelial cells P = 0.009.  Ref: 12839674 Zhonghua Bing Li Xue Za Zhi, 2003
- A significant positive correlation was found between VEGF C  [ **VEGFC** ]  expression and VEGFR 3  [ **FLT4** ]  expression in cancer cells P <.  Ref: 12875690 Chin Med J (Engl), 2003
- We measured the gene expression of VEGF ligands VEGF A, VEGF B, VEGF C  [ **VEGFC** ] , and VEGF D and their receptors VEGFR 1, VEGFR 2, and VEGFR 3  [ **FLT4** ] , in normal colorectal tissues n = 20, adenomas n = 10, and in CRC n = 71 representing different Duke s stages using ribonuclease protection assay, semi quantitative relative reverse transcriptase polymerase chain reaction, together with the pattern of their expression by immunohistochemistry.  Ref: 12754739 J Pathol< MedlineTA>J Pathol, 2003
- Vascular endothelial growth factor C  [ **VEGFC** ]  and vascular endothelial growth factor receptor 3  [ **FLT4** ]  expression in squamous cell carcinomas of the head and neck.  Ref: 12784238 Head Neck, 2003
- The VEGFR 3  [ **FLT4** ]  specific mutant form of VEGF C  [ **VEGFC** ]  called VEGF C156S lacks blood vascular side effects but is sufficient for therapeutic lymphangiogenesis in a mouse model of lymphedema.  Ref: 12888864 Thromb Haemost, 2003
- RESULTS Of the 76 NSCLC cases, 55 were VEGF C  [ **VEGFC** ]  positive and 40 were VEGFR 3  [ **FLT4** ]  positive in cancer cells.  Ref: 12875690 Chin Med J (Engl), 2003
- Immunohistochemistry in 18 HNSCC specimens assigned strong to mediate VEGF C  [ **VEGFC** ]  IR and minor VEGFR3  [ **FLT4** ]  IR to tumor cells and strong VEGF C  [ **VEGFC** ]  and VEGFR3  [ **FLT4** ]  IR to tumor surrounding vessels.  Ref: 12784238 Head Neck, 2003
- METHODS Samples of 76 NSCLC cases with the neighboring noncancerous tissue were studied using anti VEGF C  [ **VEGFC** ] , VEGFR 3  [ **FLT4** ]  and CD34 antibodies.  Ref: 12839674 Zhonghua Bing Li Xue Za Zhi, 2003
- Examination of the VEGF C  [ **VEGFC** ]  specific receptors VEGFR 2 KDR Flk 1 and VEGFR 3  [ **FLT4** ]  Flt 4  [ **FLT4** ]  demonstrated intense endothelial immunoreactivity for VEGFR 2, as well as VEGFR 2 and 3 expression on the majority of neoplastic cells, suggesting a possible role in autocrine paracrine neoplastic growth regulation.  Ref: 12861060 Mod Pathol, 2003
- These data demonstrate, for the first time, the expression of VEGFR 3  [ **FLT4** ]  and VEGF C  [ **VEGFC** ]  on tissue DC, which implicate a novel potential relationship between lymphangiogenesis and leukocyte trafficking in the eye.  Ref: 12819011 Am J Pathol, 2003
- The expression and levels of the VEGF C  [ **VEGFC** ]  and VEGF D cytokines, the VEGF receptors VEGFR 2 and VEGFR 3  [ **FLT4** ] , and newly described lymphatic endothelial markers, LYVE 1, Prox 1, podoplanin and 5 nucleotidase were assessed.  Ref: 12851706 Int J Oncol, 2003
- The broad expression of VEGF C  [ **VEGFC** ]  and VEGFR3  [ **FLT4** ]  in HNSCC suggests involvement in tumor lymph angiogenesis and vascular angiogenesis, promoting tumor growth and propagation of cancer cells.  Ref: 12784238 Head Neck, 2003
- Because HNSCC preferentially metastasizes to regional lymph nodes, we investigated the expression of VEGFR3  [ **FLT4** ]  and its ligand VEGF C  [ **VEGFC** ]  in head and neck squamous cell carcinomas by semiquantitative RT PCR 4 HNSCC cells lines and 6 HNSCC specimens and by immunohistochemistry 18 HNSCC specimens.  Ref: 12784238 Head Neck, 2003
- CONCLUSIONS In NSCLC, VEGF C  [ **VEGFC** ]  and VEGFR 3  [ **FLT4** ]  are related to the lymphangiogenesis, angiogenesis, and occurrence and development of lung cancers.  Ref: 12875690 Chin Med J (Engl), 2003
- METHODS Seventy six NSCLC samples were stained for VEGF C  [ **VEGFC** ] , VEGFR 3  [ **FLT4** ]  and CD34 with immunohistochemical methods.  Ref: 12875690 Chin Med J (Engl), 2003
- Novel Expression of Vascular Endothelial Growth Factor Receptor VEGFR 3  [ **FLT4** ]  and VEGF C  [ **VEGFC** ]  on Corneal Dendritic Cells.  Ref: 12819011 Am J Pathol, 2003
